# Overview of Mitigation Programs for Cattle Diseases in Austria

**DOI:** 10.3389/fvets.2021.689244

**Published:** 2021-06-15

**Authors:** Franz-Ferdinand Roch, Beate Conrady

**Affiliations:** ^1^Department for Farm Animals and Veterinary Public Health, Institute of Food Safety, Food Technology and Veterinary Public Health, University of Veterinary Medicine, Vienna, Austria; ^2^Department of Veterinary and Animal Sciences, Faculty of Health and Medical Sciences, University of Copenhagen, Frederiksberg, Denmark; ^3^Complexity Science Hub Vienna, Vienna, Austria

**Keywords:** animal health law, bluetongue, bovine viral diarrhea, enzootic bovine leucosis, eradication, control program, infectious bovine rhinotracheitis/infectious pustular vulvovaginitis

## Abstract

**Background:** The non-mandatory regulation of animal diseases at the European Union (EU) level enables member states to implement mitigation programs based on their own country-specific conditions such as priority settings of the governments, availability of financial resources, and epidemiological situation. This can result in a heterogeneous distribution of mitigation activities and prevalence levels within and/or between countries, which can cause difficulties for intracommunity trade. This article aims to describe the past, current, and future mitigation activities and associated prevalence levels for four animal diseases, i.e., enzootic bovine leukosis (EBL), infectious bovine rhinotracheitis/infectious pustular vulvovaginitis (IBR/IPV), bovine viral diarrhea (BVD), and bluetongue disease (BT) for Austria. Over a period of 40 years (1978–2020), regulations concerning EBL, IBR/IPV, BVD, and BT were retraced to analyze the changes of legislation, focusing on sampling, testing, and mitigation activities in Austria, and were linked to the collected diagnostic testing results. The study results clearly demonstrate the adoption of the legislation by the Austrian governments in dependency of the epidemiological situations. Furthermore, our study shows that, related to the forthcoming Animal Health Law on April 21, 2021, Austria has a good initial situation to achieve disease-free status and/or free from infection status based on the current available epidemiological situation and previously implemented mitigation activities. The study results presented here are intended to contribute to a better comparison of the eradication status across European countries for cattle diseases by providing information about the mitigation activities and data of testing results over a period of 40 years.

## Introduction

The new European Union (EU) Animal Health Law [Regulation (EU) 2016/429] (1) will be enforced on the April 21, 2021, and cover five categories (A–E) listed in Article 9 as follows:

A: “[…] diseases that do not normally occur in the Union and for which immediate eradication measures must be taken as soon as they are detected […]”
B: “[…] diseases which must be controlled in all member states with the goal of eradicating them throughout the Union […]”
C: “[…] diseases which are of relevance to some member states and for which measures are needed to prevent them from spreading to parts of the Union that are officially disease-free or that have eradication programmes for the listed disease concerned […]”
D: “[…] diseases for which measures are needed to prevent them from spreading on account of their entry into the Union or movements between member states […]”
E: “[…] diseases for which there is a need for surveillance within the Union […]”

The allocation of animal diseases is set out in the corresponding Commission Implementing Regulation (EU) 2018/1882 (2). Besides the diseases listed in categories A and B, for which Union-wide regulations are implemented, there are animal diseases with no or limited mandatory regulations listed in categories C–E such as bluetongue disease^1^ (BT), epizootic hemorrhagic disease^2^, anthrax^2^, surra^2^, paratuberculosis^3^, Q fever^3^, infectious bovine rhinotracheitis/infectious pustular vulvovaginitis^1^ (IBR/IPV), bovine viral diarrhea^1^ (BVD), bovine genital campylobacteriosis^2^, trichomonosis^2^, and enzootic bovine leukosis^1^ (EBL) (2).

No or limited mandatory regulation of these cattle diseases at the EU level enables researchers to implement mitigation programs based on country-specific conditions such as priority settings of the governments, availability of financial resources, epidemiological situation such as the level of prevalence, and the importance of export for the national economy. This results in a heterogeneous distribution of mitigation activities and prevalence levels within and/or between countries. The heterogeneous distribution of mitigation activities can cause difficulties for intracommunity trade, as trade activities with livestock can introduce infectious agents into countries that are free from disease. Based on this background, the COST (European Cooperation on Science and Technology) Action “SOUND control” (CA17110) was initiated to give an overview of the different control and mitigation programs and enable a comparison between the member states (3).

This article aims to describe the past, current, and future mitigation activities and associated prevalence of the four cattle diseases in Austria (i.e., EBL, IBR/IPV, BT, and BVD), categorized as C+D+E according to the Commission Implementing Regulation (EU) 2018/1882, and to evaluate the potential effects of the forthcoming Animal Health Law on April 21, 2021, for Austrian legislation. The study presented here addresses the lack of information regarding the mitigation activities for these four animal diseases and associated eradication status in Austria over a 40-year period. The study presented here is intended to contribute to a better comparison of implemented mitigation activities and associated eradication status across the European countries.

## Materials and Methods

Laws, ordinances, and official veterinary edicts concerning EBL, IBR/IPV, BVD, and BT were retraced from 1978 to analyze the historical development and changes of legislation, focusing on sampling, testing, and mitigation activities. All versions of the legislative documents used for the study presented here are in the public domain in the Austrian legal information system (RIS, www.ris.bka.gv.at). The Austrian ordinances and laws used for this study are referenced as “AL + a consecutive number” (see the associated references in the Supplementary Material). The full references are provided in the Supplementary Material due to the large number of different applied ordinances and laws over time in order to describe the historical development of the mitigation activities for the four cattle diseases. Information concerning European legislation was obtained from EUR-Lex (eur-lex.europa.eu).

The historical development of the EBL status is based on Commission Decision 1999/465/EC (i.e., versions from 1999 to 2002) and/or 2003/467/EC (i.e., versions from 2003 to 2020). The historical evolution of freedom from IBR/IPV including additional guarantees is obtained from Commission Decision 93/42/EEC (i.e., versions from 1993 to 2004) and/or 2004/558/EC (i.e., versions from 2004 to 2020). Animal population data were collected from the Green Report (GR; i.e., annual reports, describing the situation of Austria's agriculture and forestry) for the period 1979–2019 (4). Numbers of tested animals, positively tested animals, and affected livestock focusing on EBL, IBR/IPV, and BTV were extracted from the GR for the period between 1979 and 1997, or from the Annual Veterinary Report (AVR) for the period 1998 to 2019 (4, 5). The analyzed BVD data were extracted from the AVR and by using other sources such as upon request of the last author to the governments of all federal states via an Excel file (see all sources listed in the Supplementary Material). All figures were created with R 3.6.3 and GQIS 3.6.2-Noosa (6, 7). All data collected for the four cattle diseases, i.e., EBL, IBR/IPV, BVD, and BT, for Austria regarding the number of tested animals, tested bulk milk, positively tested animals, affected livestock, and changes in the sample size associated with changes of law over a 40-year period are provided in **Figures 2–8** and in Supplementary Tables 1–3 and Supplementary Figures 1, 2.

## Results

### Demographic Data for the Cattle Sector in Austria

**Figure 1:**
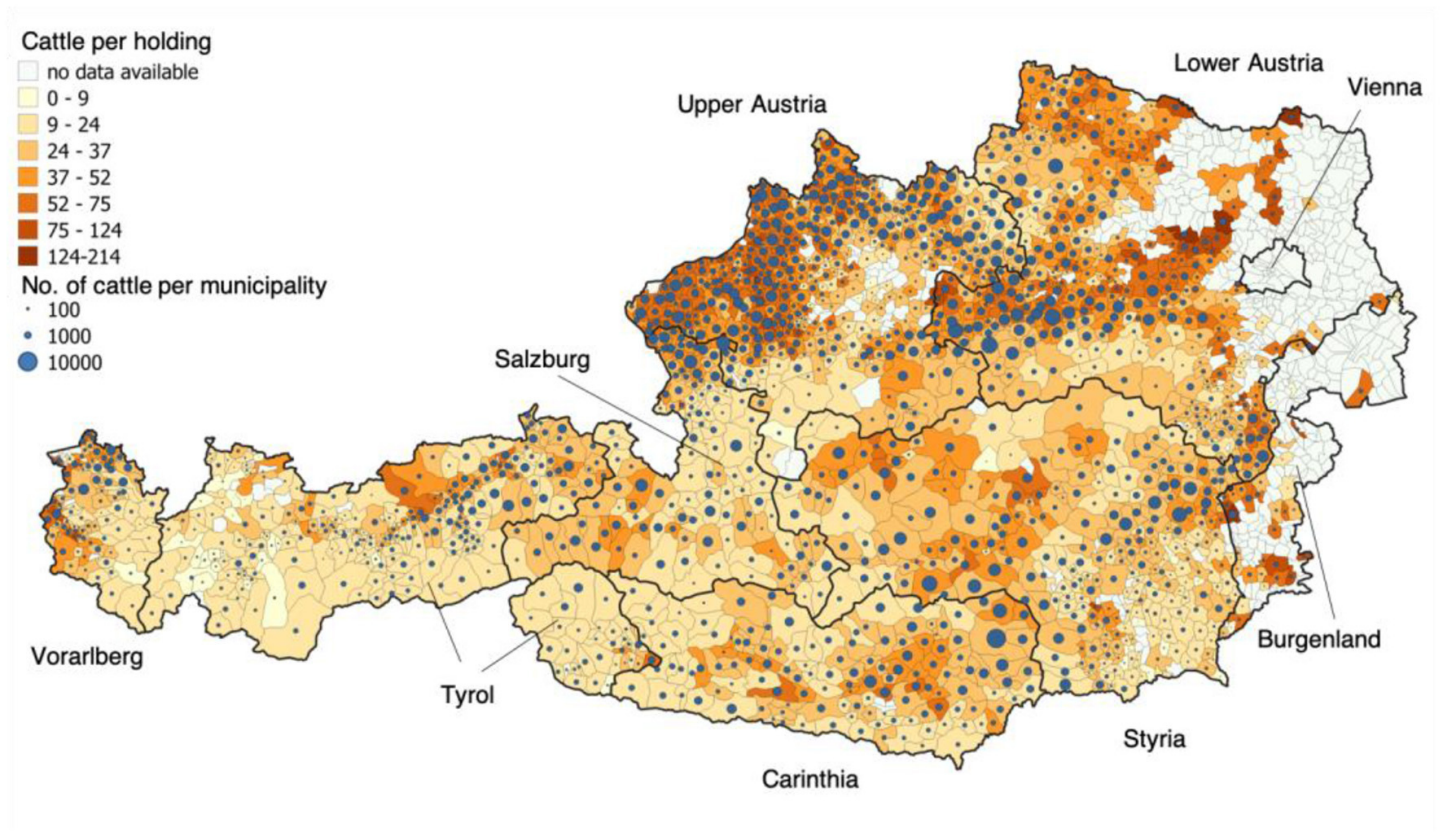
Regional distribution of Austria's cattle population [data from 2017 (9)]. The colors of the municipalities shown depend on the average number of cattle per holding. The size of the blue dots represents the absolute cattle number per municipality. The recent available data from the year 2017 were used for this figure (4).

The added gross value of Austrian agriculture amounted to €7.48 billion in 2019, of which €2.17 billion can be assigned to cattle (GR 2020) [note: ~1.84 million cattle located in 55,751 cattle holdings (mean herd size: 33 cattle/herd) (8); most of them are located in Upper and Lower Austria; Figure 1]. The export volume totaled €1.90 billion for cattle in 2019 (i.e., milk and milk products approx. €1,260 million, cattle: €88 million, beef: €450 million), while the import volume was €1.17 billion (10, 11). In total, Austria exported 56,173 cattle (10,410 for direct slaughter) and 45,423 calves (672 for direct slaughter) in 2019 (12), whereby Austria imported 97,257 cattle (95,455 for direct slaughter) and 4,071 calves (3,565 for direct slaughter) for the same year (12). Thus, from an economic point of view, a disease-free cattle population is highly important for the livestock trade in Austria.

### Enzootic Bovine Leukosis

**Figure 2:**
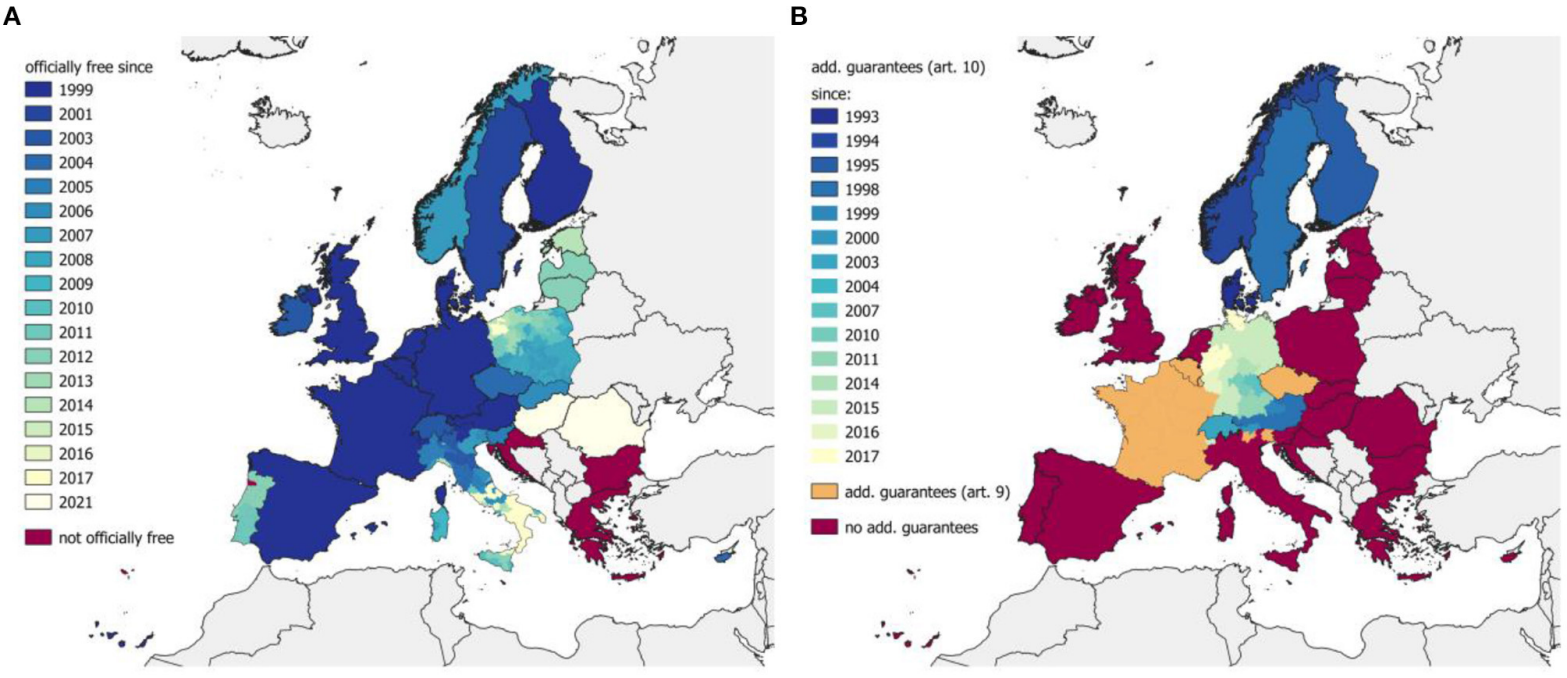
Historical development of freedom of EBL **(A)** and IBR/IPV **(B)**. The EU grants the member states an officially EBL-free status and additional guarantees for IBR based on Council Directive 64/432/EEC. The concerned member states are listed in the Commission Decision 2003/467/EC (31) [since 2003, before that in 1999/465/EC (32)] for EBL and in Commission Decision 2004/558/EC (33) [since 2004, before that in 93/42/EEC (34)]. Switzerland and Norway have separate agreements. Information about the EBL status of Switzerland was collected from Appendix 2(I)(B)(5) of Annex 11 to the Agreement between the EU and the Swiss Confederation on trade in Agricultural Products (2004/78/EC) (35) and Norway on EFTA Surveillance Authority Decision 28/07/COL (36). IBR/IPV information for Switzerland was obtained from Appendix 2(I)(B)(6) of Annex 11 to the Agreement between the EU and the Swiss Confederation on trade in Agricultural Products (2004/78/EC) (35) and Norway on EFTA Surveillance Authority Decision 74/94/COL (37).

EBL is an infectious disease caused by the bovine leukemia virus (BLV), a retrovirus and oncogenic member of the *Deltaretrovirus* genus (13, 14). Infections are in most cases subclinical, but ~30% of infected cattle develop a persistent lymphocytosis (15) caused by B-cell expansion (16); fewer than 5% of the infected animals develop tumors (lymphosarcoma), which are typically observed in animals older than 3 years (15, 17, 18). Clinical signs depend on the localization of the tumors and include lymphadenopathy, inappetence, digestive malfunction, loss of weight, debility, and sometimes neurological symptoms (19). The transmission of BLV can be vertical, by *in utero* infection or colostrum intake, or horizontal, by direct animal contact, oral or parenteral viral uptake, iatrogenic (e.g., needles, rectal palpation), or by hematophagous flies (20–25). Economic impacts and consequences for animal welfare in affected herds (independently if cases are clinical or subclinical) are reduction of milk production, lower conception rates, and a higher susceptibility to other infectious diseases such as mastitis, diarrhea, or pneumonia (26–29). Some European countries started control measures against EBL decades ago [e.g., Denmark in 1959, Finland in 1966; (30)]; thus, most of today's EU members are officially EBL-free (Figure 2A).

**Figure 3:**
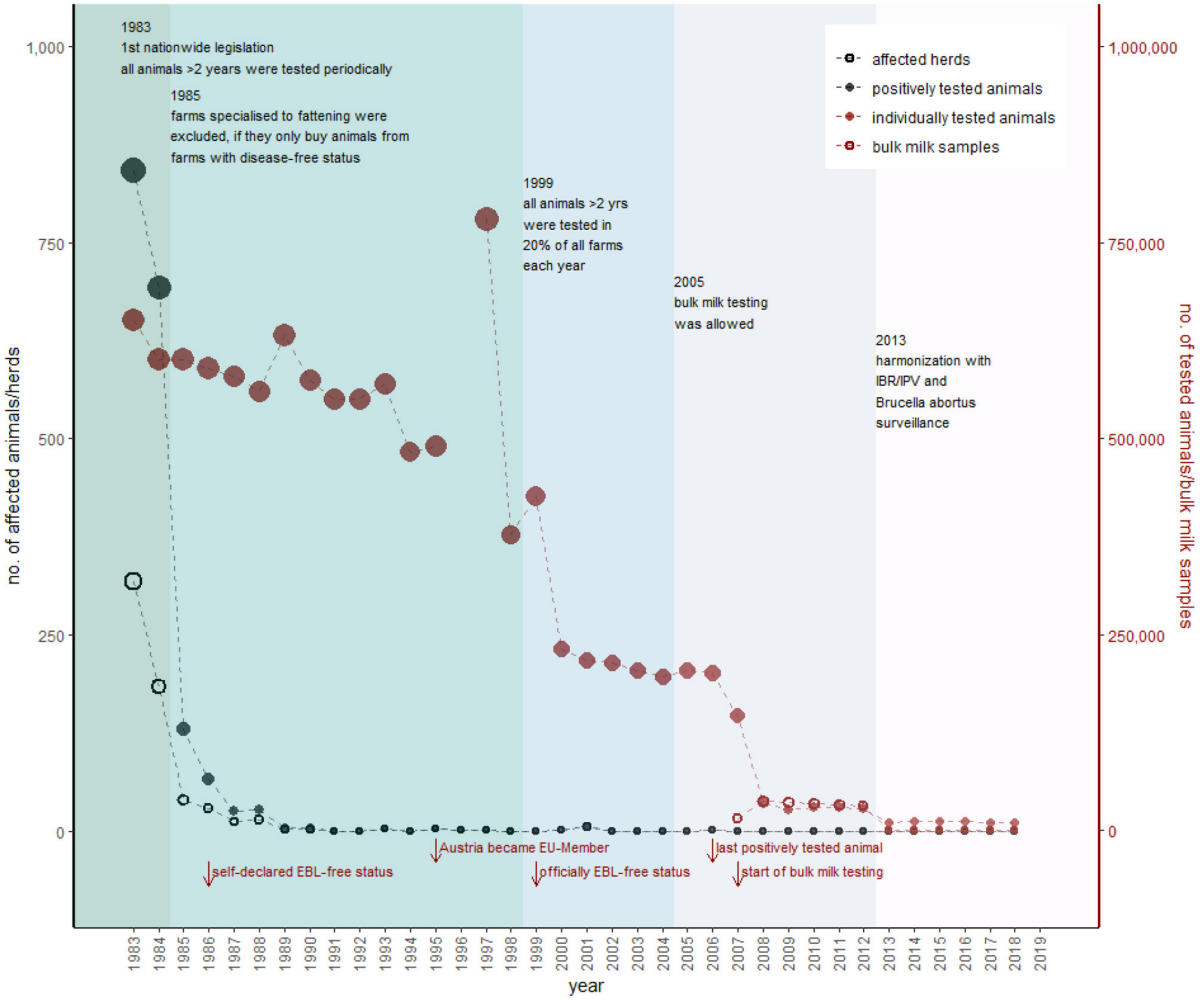
Overview of the historical development of EBL control in Austria. Black dots represent the number of positively tested animals (filled) and number of herds with at least one positively tested animal (empty), according to the primary *y*-axis. The red dots show the number of individually tested animals (filled) and number of herds tested via bulk milk (empty), according to the secondary *y*-axis (detailed data are provided in Supplementary Table 1). The most important changes in legislation with consequences for the sampling strategy represent the different colors within the figure. Red arrows show further essential events regarding EBL in Austria.

The control of EBL in Austria started in 1979 with a voluntary eradication program, which was financially promoted by the federal and state governments (GR 1980), followed by a national compulsory eradication program of EBL in 1982 (AL1). According to the accompanying legislation, all animals older than 2 years had to be periodically tested at intervals of 21–27 months (AL1). This resulted in ~600,000 animals tested each year (Figure 3). The sampling was combined with the sampling for the control of *Brucella abortus* (Morbus Bang), which was established in the year 1957 (AL2). All animals reacting to any of the tests (positively tested animals) had to be slaughtered (AL1), including (i) cattle with a positive antigen test, (ii) cattle >6 months with a positive antibody test, (iii) cattle >6 months with three inconclusive antibody tests in a row (note: exceptions existed for pregnant animals listed in a breed register or of special endangered breeds), and (iv) calves <6 months born or suckled from a positively tested animal (AL1). Thus, EBL vaccination is still forbidden in Austria (AL1, AL3).

The number of detected positively tested animals decreased from 842 (in 318 holdings) in 1983 to 26 (in 14 holdings) in 1985 (Figure 3). In 1986, Austria declared all its federal states disease-free, based on the definition of the law of 1982 (GR 1986). In detail, a federal state achieved the EBL-free status when all livestock in the federal state was tested at least two times, and the proportion of positively tested animals was <0.2%, or the proportion of farms with at least one positively tested animal was <0.5% during the second testing (AL1).

However, the officially EBL-free status in Austria (according to Commission decision 1999/465/EC) (38) was achieved in 1999, after EBL was incorporated to EU directive 64/432/EEC (39). In this context, the sampling plan was adapted and all animals older than 2 years were tested in 20% of all livestock holdings each year (AL4). This sampling strategy decreased the annual number of tested cattle from 400,000 in 1999 to 200,000 in 2000. In 2007, the nationwide already established bulk milk testing was applied (AL5), and ~35,000 holdings were tested each year onward from 2007 (AVR 2007). Consequently, the number of individually tested animals decreased to 30,000 per year, compared to previously 600,000 tested animals (Figure 3). A further reduction in the sample size occurred in 2013 because of harmonization of the control of EBL, IBR/IPV, and *B. abortus* in one law and sampling plan (AL6). This annual sampling plan should ensure that <0.2% of livestock are infected with a confidence rating of 99% (AL7) and is in accordance with EU directive 64/432/EEC Annex D Chapter 1 F. This implied the testing of ~11,000 animals in 1,300 holdings and testing of bulk milk samples (with a maximum of 50 lactating cows per bulk milk) of 1,300 additional farms per year (AL7) (Figure 3).

The last positively tested animal was found in Austria in 2006 (AVR 2006), without consequences for the disease-free status. The disease-free status remains as long as 99.8% of livestock has a disease-free status. For stocks that lose their declared disease-free status, it is forbidden to market animals, participate in shows, introduce new animals into the herd, use animals for the recovery of semen or embryos, or involve animals in mating (AL3). To regain the EBL-free status, all positively tested animals have to be removed, and after a monitored disinfection supervised by the governments, all remaining animals >6 months have to be tested negative twice within an interval of at least 6 months (AL3).

### Infectious Bovine Rhinotracheitis/Infectious Pustular Vulvovaginitis

IBR/IPV is an infectious disease caused by the bovine herpesvirus (BHV-1), of the subfamily *Alphaherpesvirinae*. In most cases, the virus causes the respiratory disease IBR, which affects the upper respiratory tract as rhinitis and tracheitis. The genital form shows up as balanoposthitis (infectious balanoposthitis IBP) in males and vulvovaginitis (IPV) and abortion in female animals. The transmission mainly occurs through direct animal contact via respiratory, ocular, or genital secretions or through the semen of infected bulls (40). Economic losses are caused by abortion, fertility disorders, decrease in milk production, and further in costs for infection control measures and trading restrictions (41, 42). Some European countries eradicated IBR/IPV and have additional guarantees since the 1990s, but most EU member states still have IBR/IPV present in their livestock (Figure 2B).

Austria's control of IBR started in 1988, after the government estimated a prevalence between 0.8 and 1.0%, with a nationwide voluntary eradication program (GR 1987 and 1988). During the first 2 years of this voluntary program, ~9,000 positively tested animals were culled (GR 1990). In 1990, national compulsory eradication of IBR was established (AL8). The IBR/IPV sampling was conducted simultaneously with the EBL and *B. abortus* (AL9). Similar to EBL, all cattle older than 2 years had to be tested in a period of between 21 and 27 months, and positively tested animals had to be slaughtered (AL9). Therefore, the vaccination against IBR is still prohibited (AL8, AL3).

**Figure 4:**
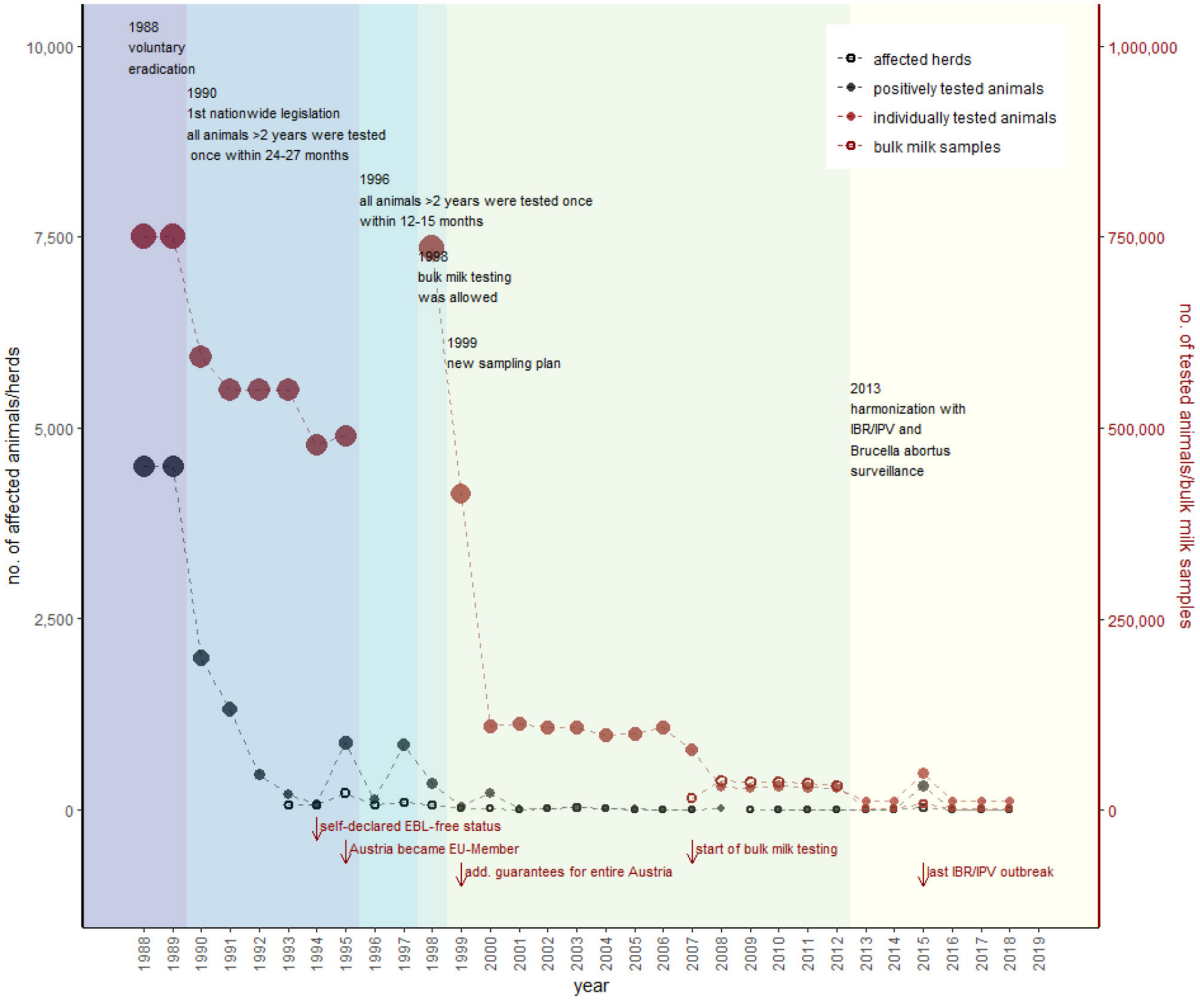
Overview of the historical development of IBR/IPV control in Austria. Black dots represent the number of positively tested animals (filled) and number of herds with at least one positively tested animal (empty), according to the primary *y*-axis. The red dots show the number of individually tested animals (filled) and number of herds tested via bulk milk (empty), according to the secondary *y*-axis (detailed data are provided in Supplementary Table 2). The most important changes in legislation with consequences for the sampling strategy represent the different colors within the figure. Red arrows show further essential events regarding IBR/IPV in Austria.

The number of positively tested animals decreased from 1,989 in 1990 to 72 in 1994. A self-declared IBR/IPV-free status for all federal states in Austria was introduced in the year 1994 (GR 1994) (Figure 4). In 1995, the number of detected animals increased to 847, primarily caused by an increase in trade activities because Austria became a member of the EU (GR 1995). Consequently, the period between two samplings was reduced to a 12- to 15-month interval in 1996 (AL10). The sampling plan changed again in 1999 (AL11) to meet the requirements of additional guarantees according to the EU directive 64/432/EEC in 2007, when bulk milk testing was established nationwide (AVR 2007), and in 2013, when IBR/IPV, EBL, and *B. abortus* control was harmonized (AL6), and sample size had to ensure that <0.2% of livestock herds were infected (confidence rating of 99%) (AL7). The EU has granted additional guarantees for most Austrian regions since 1998 (compared to Figure 2B) and for the whole of Austria since 1999 (43).

The last IBR/IPV outbreak was in January 2015 when an infection was detected during an export examination (AVR 2015). As a reaction to this outbreak, the Austrian government has tested 15,823 animals in addition to the already included 32,559 cattle in the surveillance program. In total, 313 positively tested animals in 26 herds were detected and removed (Figure 4). The additional guarantees remained unaffected (AVR 2015). The consequences for holdings losing their status and conditions for regaining it are the same as for EBL, but the interval between the two tests of all remaining animals is 4 weeks instead of 6 months (AL 3).

### Bovine Viral Diarrhea

BVD virus (BVDV) is a pestivirus within the family Flaviviridae, belonging to the genus *Pestivirus*. BVDV can be divided into two main genotypes, BVDV-1 and BVDV-2, and both genotypes can also be classified by biotyping in cytopathogenic (cp) and non-cytopathogenic (ncp) types (44–46). Because of lifelong shedding of large amounts of virus, persistently infected (PI) animals are the primary source of BVDV. Persistent BVDV infections can arise by (i) transmission of ncp BVDV from an already PI cow to the fetus (i.e., PI dam always delivers a newborn PI calf, and thus, the removal of such animals from the herd is essential to interrupt the infection cycle), or (ii) acute infection of susceptible pregnant cows with ncp BVDV between ~90 and 120 days of gestation. During this period, the fetus is not able induce an immune response against BVDV. If the fetus survives, the newborn calf will be PI and is usually unable to develop virus-specific antibodies (AB) to BVDV (referred to as immunotolerant) (47, 48). Because of the short infection period, most acute infections will not establish PI animals in the subsequent generation (49). Seronegative cattle will become acutely (transiently) infected after contact with a PI animal (49, 50) and produce AB against BVDV within ~2–4 weeks (also described as seroconversion) (51). The most frequently observed symptom of animals after BVDV infection is growth retardation, but the cattle can also be clinically healthy (52). The latter is important from an epidemiological point of view and the main reason to perform diagnostic tests to identify PI animals. BVDV is an important infectious agent in the cattle population and has a global economic impact both through production losses such as reproductive dysfunction and costs of mitigation activities (53–59).

The mitigation of BVD in Austria can be distinguished into two phases. The first time period is between the years 1996 and 2004, when several federal states implemented voluntary eradication programs (Lower Austria 1996, Styria 1998, Tyrol and Vorarlberg 1999, Upper Austria 2000) (57, 60–63). Because of relatively high seroprevalences, Tyrol and Vorarlberg focused on individual antigen testing to detect PI animals in beef and dairy herds, whereas federal states with lower seroprevalences (i.e., Lower Austria and Styria) used bulk milk testing for screening of dairy herds, followed by individual testing (milk or blood). In all federal states, it was a strict non-vaccination strategy combined with the elimination of PI animals. The second time period started in 2004, when the nationwide compulsory eradication and control program was established (AL13). Although revised several times, the conditions for receiving the officially free status are still the same (AL14). However, the conditions for keeping the status were updated in 2018 and allowed testing based on a sampling plan for non-dairy herds (**Figure 6**) (AL15). The regulation applies to all farms except fattening farms without breeding. For the movement of animals from the holding to locations with possible contact to other cattle (e.g., market, shows, breeding, community pastures), individual testing of the affected animals is mandatory. In general, animals consigned directly to the slaughterhouse were excluded from testing according to regulation. Further exclusions depended on status of the holding, age of the animal, pregnancy status, period to the last diagnostic testing, how long the affected animal had been kept in the holding, and in which federal state the holding was located (see detailed description in Supplementary Figure 1). These exemptions are valid for a period of 1 year and can be extended by reapplication by the federal states annually. In order to get the exemption from testing, the following requirements have to be fulfilled: (i) the federal state should not have had any new BVD outbreaks within the previous 2 years; (ii) the proportion of officially-free herds in the federal state is ≥95%; (iii) tests were performed properly until the beginning of the exemption; and (iv) a proper surveillance program exists (AL15).

**Figure 5:**
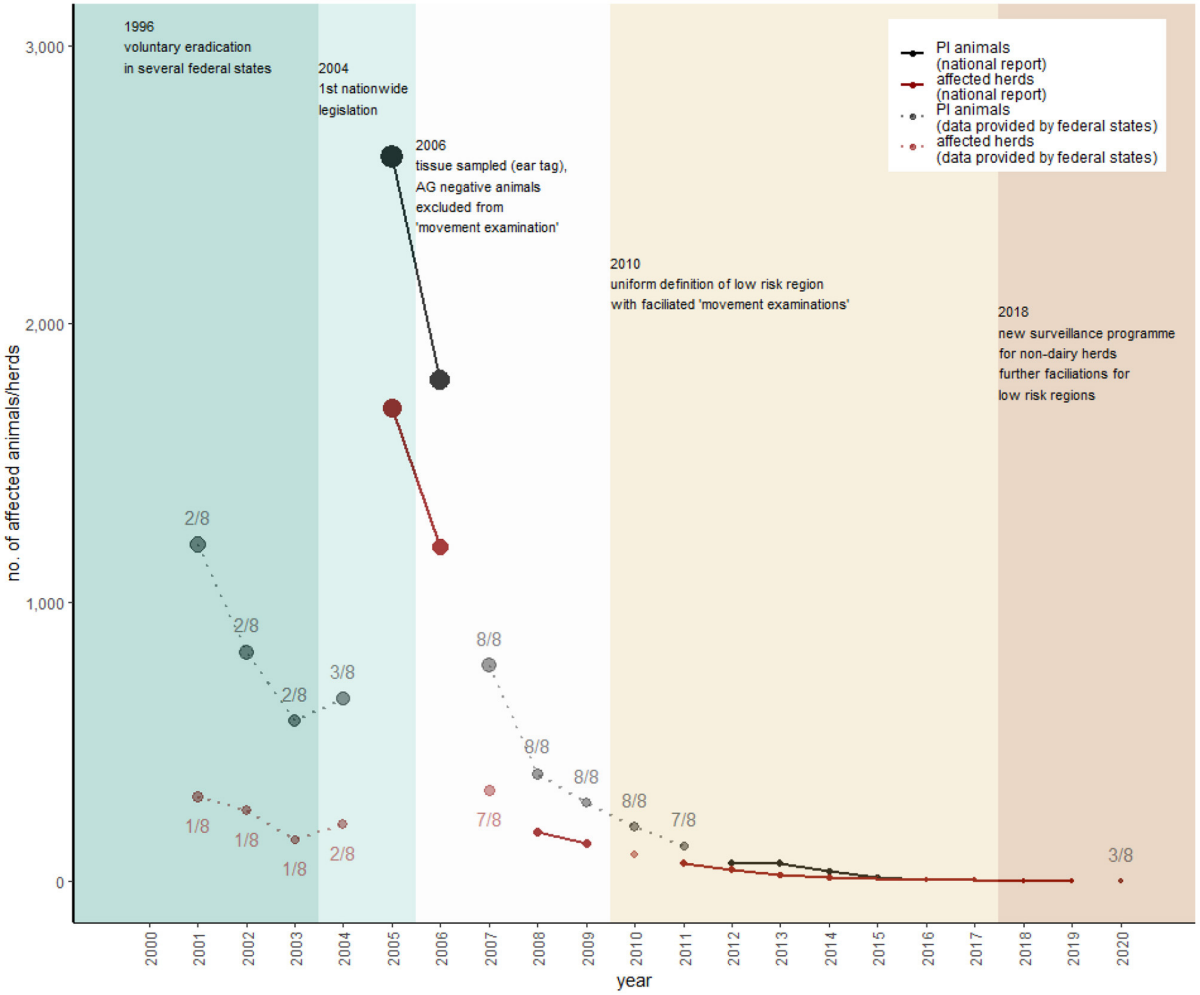
Overview of the historical development of BVD control in Austria. National report data of persistently infected (PI) animals are represented as black dots and herds with PI animals as dark red dots. These data are not consistently available over the period. Missing data were supplemented with federal state data upon our request, represented as lighter dots. To provide an estimation of how representative these federal state data are, the numbers of federal states (out of eight) are included in each data point as a number (detailed data of the individual federal states are presented in Supplementary Figure 2). The most important changes in legislation with consequences for the sampling strategy represent the different colors within the figure.

**Figure 6:**
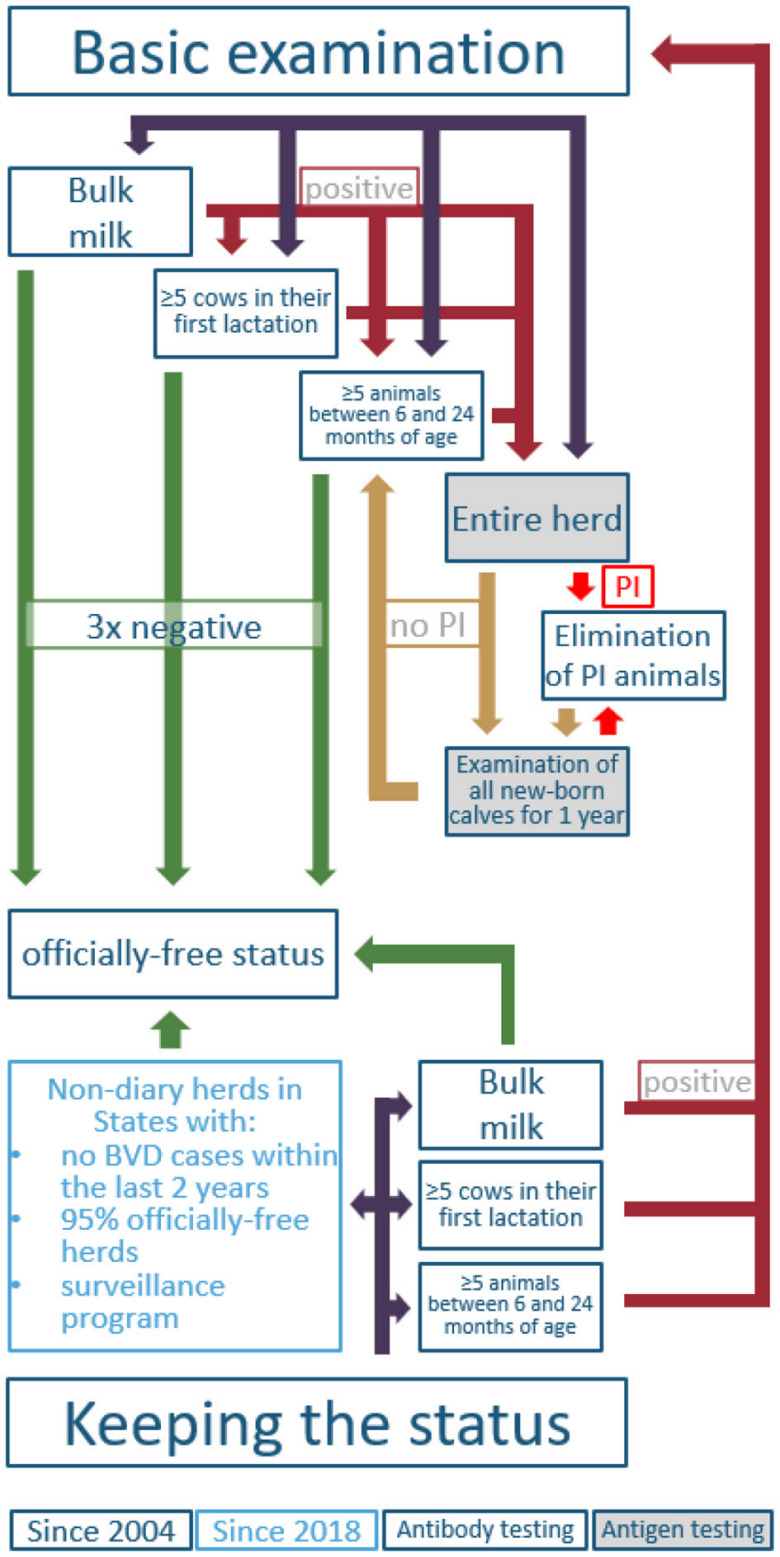
Possible ways to gain and keep a BVD-free status for cattle holdings in Austria, according to the BVD legislation (AL14). PI, persistently infected animal.

Figure 5 shows that data regarding BVD testing are not publicly available for every year. In 2005, ~2,600 PI animals were detected and, so far (state of 26 March 2021), the last three PI animals in the Austrian cattle population were detected in 2017 (AVR 2017). In 2011, 92% of all holdings, subjected to the legislation, were officially BVD-free (AVR 2011). Since then, no detailed data have been published, and veterinary reports have annually declared that Austria's cattle holdings, subjected to the legislation, are “nearly entirely officially BVD-free” (AVR 2012-2018). Figure 6 shows that there are several ways to gain and keep a BVD-free status for cattle holdings in Austria (AL15), but the most common way is testing of bulk milk regarding BVDV antibodies.

### Bluetongue Disease

BT is caused by bluetongue virus (BTV), a member of the genus *Orbivirus* within the family *Reoviridae* (64), which is assigned to 28 different serotypes (65–68). Sheep and some wild ruminants are the most clinically affected species, showing oral erosions and ulcers, lameness and coronitis, weakness and depression, and facial edema, whereas clinical infections in cattle were not observed until 2006. In 2006, BTV serotype 8 was introduced to northern Europe for the first time (67–69). Besides recurring outbreaks in Cyprus (since 1924) (70) and few outbreaks in the late 1950s in Spain and Portugal (BTV-10) and 1979/1980 in Greece (BTV-4) (71, 72), BT was considered to be an exotic disease in Europe until 1998, when it was introduced to the Mediterranean Basin (69, 72). The introduction of BTV-8 to northern Europe in 2006 showed that not only *Culicoides imicola*, the main vector of BTV in the Mediterranean Basin, but midges of the *Culicoides obsoletus* complex (including *Culicoides dewulfi*), widespread in northern and central Europe, are very effective at transmitting BTV between host ruminants (70, 73–76). In the outbreak of 2006, BTV-8 caused a high rate of abortions, still births, and fetal malformations, which indicated a (subsequently confirmed) transplacental infection (77–82). Direct horizontal transmission was rarely described (65, 83–85). Economic impacts (especially in epidemic situations) are reduced fertility, dead animals, decreased milk production, costs for vaccines, and trading restrictions (86–88).

While EBL and IBR/IPV were eradicated in Austria, and a reintroduction through the import of infected animals is manageable, the control and the maintenance of the disease-free status for BT are more challenging because of the uncontrollable entry of infected vectors. Thus, the control measures of BT differ substantially from EBL and IBR/IPV.

In 1993/1994, BT was listed in the Austrian Animal Disease Act as a notifiable disease (AL16, AL17), and in 1996/97, the import and translocation of animals from affected regions were forbidden (AL18). According to Council Directive 2000/75/EC (89) and Commission Regulation (EC) No. 1266/2007 (90), in 2007 Austria established a nationwide surveillance program and two legislative regulations for surveillance (BTÜ-V) (AL19) and control and eradication of BT (AL20). The surveillance is fully financed by the federal government and is based on four pillars: (i) a sampling plan for serological examinations; (ii) the use of sentinel animals; (iii) the surveillance of vectors via traps, all three regulated in the BTÜ-V; and (iv) a passive surveillance by examining suspicious clinical cases. The sampling plan aims to demonstrate the absence of BT. Additionally, vector traps are used to define seasonally vector-free periods. Compared to sheep (402,658) and goats (92,504), the total stock of cattle (1,800,000 in the year 2019) is 4–19 times higher in Austria; thus, the surveillance mainly focuses on cattle.

**Figure 7:**
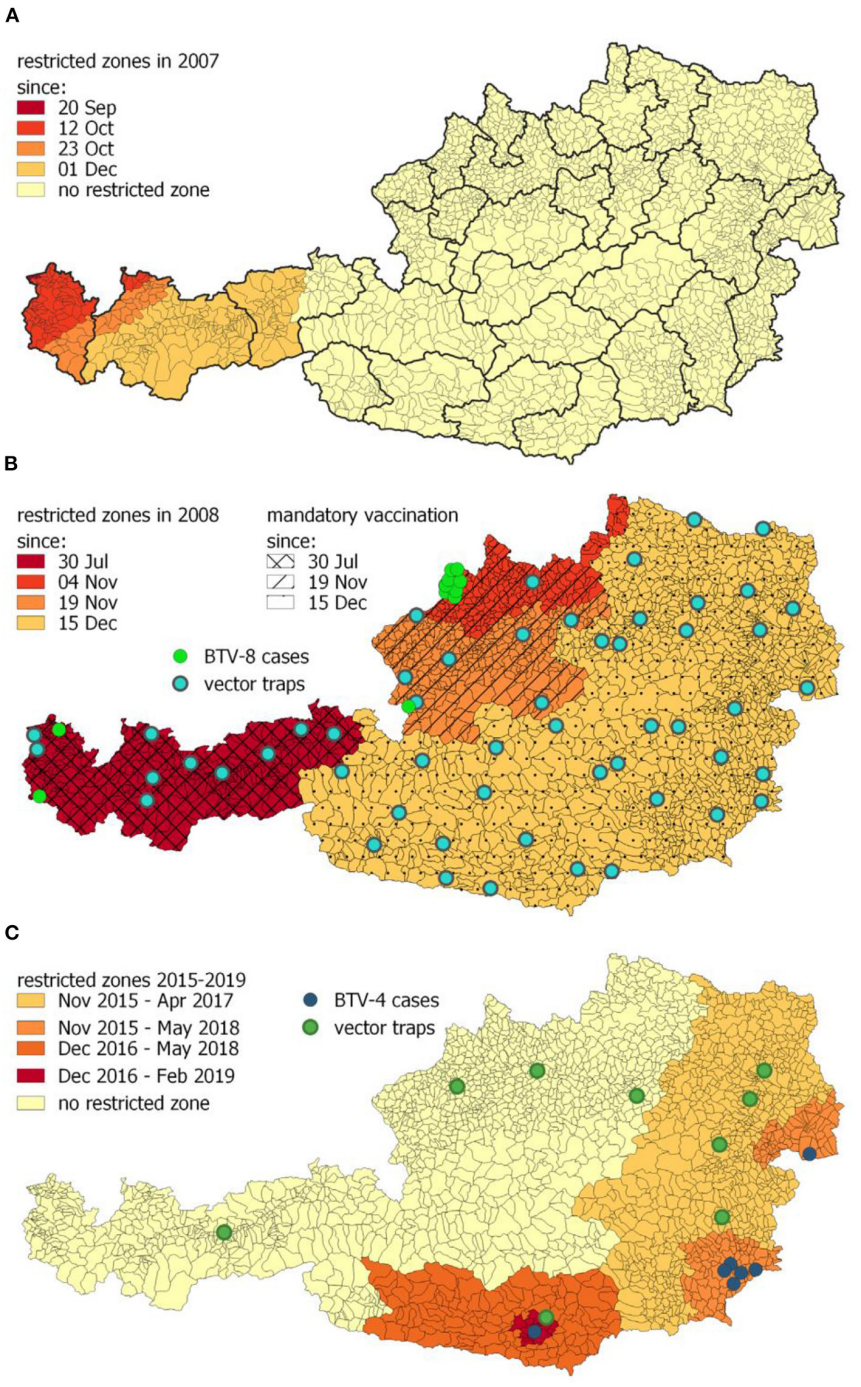
**(A)** In total, 28 sentinel regions in Austria and the stepwise established restricted zones in 2007 during the BTV-8 outbreak in southern Germany. **(B)** Distribution of the restricted zones and mandatory vaccination (hatched areas) areas, as a consequence of the BTV-8 cases in western and northwestern Austria (green dots). The restricted zones were repealed in March 2011. Locations of the 54 vector traps to analyze the distribution of *Culicoides* spp. (blue dots) in Austria. **(C)** Development of restricted zones from 2015 to 2019, as a consequence of the BTV-4 outbreaks in 2015 and 2016 (blue dots). Green dots represent the vector traps aiming to obtain information to estimate the seasonally vector-free periods (91).

**Figure 8:**
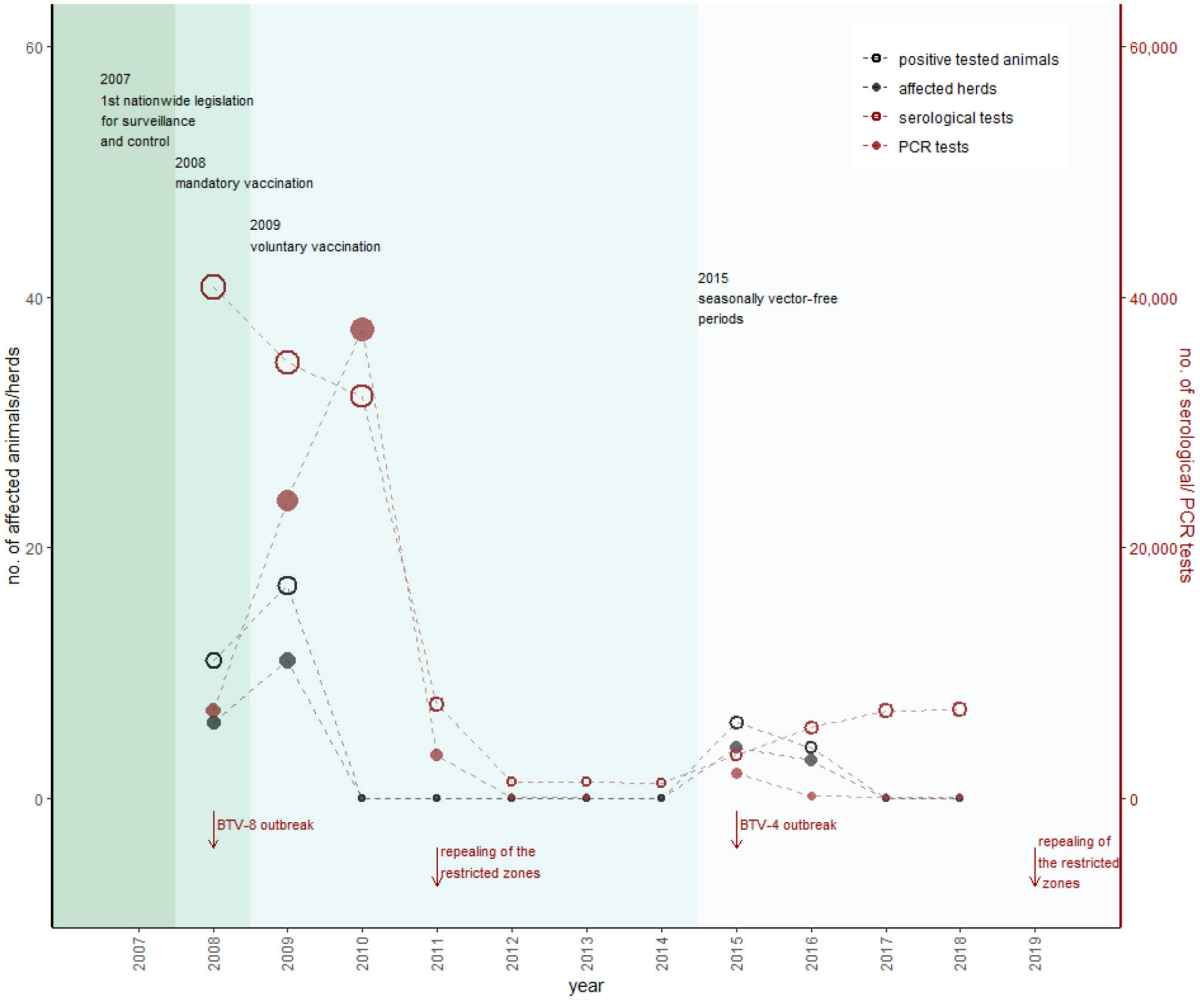
Overview of BT sampling in Austria. Black dots represent the number of positively tested animals (empty) and affected herds with at least one positively tested animal (filled), according to the left *y*-axis. Red dots show the number of serological (empty) and PCR tests (filled), according to the right *y*-axis (detailed data are provided in Supplementary Table 3). The sampling plan is designed to detect a prevalence of 5% with a 95% confidence level. The most important changes in legislation are represented by the different colors within the figure. In contrast to the other animal diseases, changes in the sampling plan are mainly caused by outbreak events (in Austria and abroad).

During the BTV-8 outbreak in northern Europe and as a consequence of detected cases in southern Germany, Austria established restricted zones [according to EU Commission Regulation (EC) No. 1266/2007] in western federal territory during 2007 (Figure 7A) (AL21-AL24). Figure 7B shows that with introduction of the mandatory vaccination, the restricted zones were extended stepwise during 2008/2009 (AL25-AL27). By the end of 2008, the whole of Austria was a uniform restricted zone, divided into 28 sentinel regions, and by the end of March 2009, 1,600,000 cattle, 344,000 sheep, and 65,000 goats were vaccinated in total (AVR 2010). In 2008, 46,503 samples were tested for antibodies (40,768 cattle, 2,820 small ruminants and 218 other species), and 8,340 samples were tested with polymerase chain reaction (PCR) (6,994 cattle, 1,293 small ruminant, and 53 other species), whereas 11 positive animals were detected (AVR 2008). In total, 17 animals were tested positive for BTV in 2009, and the last positive detection of BTV-8 in a PCR test was in March 2009 (Figure 8) (AVR 2009). The low number of cases encouraged the government to switch from a mandatory vaccination to a voluntary vaccination campaign during 2009 (AL28). Only inactivated vaccines containing certain serotypes (currently BTV-8, BTV-4 or other serotypes, if they are a part of a polyvalent formulation with BTV-8 and/or BTV-4) were used in Austria (AL28). Austria repealed all restricted zones 2 years later in March 2011 and changed to a sampling plan for a seasonal surveillance program, testing ~1,250 susceptible animals each year (Figure 8) (AVR 2012).

Because of BTV-4 outbreaks in southeastern Europe in 2014, Austria increased surveillance activities in spring 2015. Additionally, a high-risk zone in southeast Austria was implemented including monthly testing activities (AVR 2015). As a consequence of detecting BTV-4–positive cattle in November 2015, a restricted zone in eastern Austria was established, and 60 animals in each of the 28 sentinel regions were tested (AL29, AVR 2015). Figure 7C shows that this restricted zone was extended in December 2016 (AL30) when the last positive cattle were detected (AVR 2018) and was stepwise reduced until February 2019, when Austria repealed all restricted zones (Figure 7C) (AL31-AL33).

Currently, surveillance is based on the quarterly testing of 60 unvaccinated animals in each of 28 regional units. The sampling plan demonstrates a disease-free status with 95% confidence at a target of 5% prevalence (91). Additionally, nine vector traps (Figure 7C) for vector monitoring are used to determine the seasonally vector-free period (91). The traps are located in regions with the periodically longest risk for BTV transmission, based on the data of *Culicoides* spp. distribution, which were collected in 54 vector traps (Figure 7B) during the years 2008 to 2010 (AVR 2010).

Different to EBL and IBR/IPV, there is no officially BTV disease-free status for member states or individual farms. Currently, there are no restricted zones established in Austria and thus no restrictions on trade or transport of cattle (AL34). In the case of an BTV outbreak, restricted zones and transport restrictions will be set up according to EU Commission Regulation (EC) No. 1266/2007 (AL34).

## Discussion

This study shows the historical development and changes of legislation, focusing on sampling, testing, and mitigation activities for Austria, which were also linked to the collected diagnostic testing results. The study results demonstrate that the adoption of the legislation by the Austrian governments occurs in dependency of the epidemiological situations over the period. Although the study results presented here clearly demonstrate the adaption of the legislation by the Austrian governments in dependency of the epidemiological situations, the adaptation of the regulation and associated control strategy could be adjusted faster. For instance, Marschik et al. show that an adaption of the mandatory control and eradication program to risk-based surveillance for BVDV would save a lot of money for the governments and thus for the taxpayers (57). Furthermore, our study shows that, related to the forthcoming Animal Health Law on 21 April 2021, Austria has a good initial situation to achieve the disease-free status and/or free from infection status based on the current epidemiological situation and previously implemented mitigation activities.

In detail, in contrast to the official disease-free status and additional guarantees previously laid down in various regulations, the Commission Delegated Regulation (EU) 2020/689, supplementing Regulation (EU) 2016/429 (Animal Health Law), describes the conditions necessary to achieve and maintain disease-free status or the status “free from infection” for several animal diseases. Commission Delegated Regulation (EU) 2020/689 covers the requirements for *B. abortus, Brucella melitensis, Brucella suis, Mycobacterium tuberculosis* complex, EBL, IBR/IPV, Aujeszky disease, BVD, rabies, and BT, as well as for *Varroa* spp., Newcastle disease, and several diseases concerning aquaculture.

The Austrian legislation and surveillance programs for EBL and IBR/IPV do not need to be fundamentally changed to obtain the official animal disease-free status in the future. To maintain the EBL disease-free status after 5 years of freedom, a surveillance program should be implemented, which demonstrates the absence of infection by taking into account the systems of production and the risk factors, according to what the Commission Delegated Regulation (EU) 2020/689 requires in Annex IV Part III Chapter 2 Section 2 (c). Austria's EBL surveillance program is designed to detect a prevalence of 0.2% affected herds with a 99% level of confidence (AL7). Thus, to the authors' knowledge, this even meets the requirements within the first 5 years after granting disease-free status. Furthermore, the legislation contains provisions in case of an outbreak and measures for the recovery of the status, which also correspond to Commission Delegated Regulation (EU) 2020/689. Simultaneously to EBL, the surveillance of IBR/IPV can adapt after 5 years after the status was granted: The “[…] surveillance may be carried out to demonstrate yearly the absence of infection with BoHV-1, taking into account the systems of production and the risk factors identified, provided no outbreaks have been detected for 5 consecutive years following the granting of the status free from IBR/IPV in this member state or zone.” Austria's IBR/IPV surveillance program detects a prevalence of 0.2% of affected holdings with a 99% level of confidence (AL7) and thus meets the requirements of Annex IV Part IV Chapter 2 Section 2 1(b). Furthermore, vaccination is still forbidden, and the legislation contains provisions in case of an outbreak and measures for the recovery of the status, which also correspond to Commission Delegated Regulation (EU) 2020/689.

Recently, the Commission Delegated Regulation (EU) 2020/689 enables a BVD-free status for individual holdings and member states (or zones). To the authors' knowledge, Austria will make use of this opportunity, whereby a few changes in the Austrian BVD legislation perhaps would be necessary. So far, the Austrian law subjected all cattle holdings except fattening farms without breeding activities and moving animals exclusively to abattoirs. This means these holdings have not yet been able to obtain legal BVD-free status in Austria. On the other hand, the Commission Delegated Regulation (EU) 2020/689 allows these establishments to hold such a status if “all bovine animals originate from establishments free from BVD […]” (92). For the granting of the BVD-free status for member states, the regulation requires that “[…] (a) vaccination against BVD has been prohibited for kept bovine animals; (b) no case of BVD has been confirmed in a kept bovine animal for at least the previous 18 months; and (c) at least 99,8 % of the establishments representing at least 99,9 % of the bovine population are free from BVD” [Annex IV Part VI Chapter 2 Section 1 (a–c)] (92). Vaccination is forbidden, and the last PI animals were detected in Austria in 2017 (state as of 27 March 2021). The authors of this study did not receive the essential information to be able to assess whether the conditions of point (c) are fulfilled. Not all farms fall within the scope of the BVD Ordinance in Austria and are therefore covered by the sampling plan. We estimate this proportion to be ~10% of all cattle farms in Austria. However, we also know that the animals kept on these farms and originating from Austria must either come from BVD-free farms or have undergone an individual testing at animal level. Thus, we assume that it will be possible for the majority of the Austrian cattle holdings to obtain BVD-free status and that there will be no obstacles to obtaining the BVD-free status for Austria. In the future, there is a high probability that the already implemented mitigation activities without vaccination will be maintained. BVD control at a national level has been carried out without vaccination for more than 15 years, and BVDV was successfully eradicated from the cattle population. A rough estimate of the costs for a vaccination campaign would be €3.8 million for the cattle population in Austria, for an entire lifespan of a cattle population with an average lactation period of 3.91. The vaccination costs would be 10–12 times higher than the testing costs of the blood samples of the current mandatory testing of 1,242 cattle holdings (state of 2020; 10 animals per holding) and bulk tank testing based on the current implemented risk-based surveillance system to control BVDV in Austria. The benefit of the implemented control programs was that Austria is (almost) free of diseases/infections, which not only increase animal health and animal welfare but also strengthen Austria's position in the trade of cattle. For instance, Marschick et al. show that because of the implementation of the mandatory BVDV control and eradication programs, the trade of cattle increased compared to the period without compulsory BVDV control and eradication programs, and thus, a monetary gain in the trade of cattle was reached (57).

In contrast to EBL, IPR/IPV, and BVD, no general disease-free status is granted for BT. Instead, the Commission Delegated Regulation (EU) 2020/689 grants two types of status: (i) status free from infection with BTV and (ii) seasonally BTV-free. Austria is self-declared free from BT (91), and currently, no restricted zones exist. How the veterinary authority will act in the future with regard to bluetongue mitigation and what kind of disease status will be sought are unknown for the authors of the present study. However, as the Commission Regulation (EC) No. 1266/2007 has not yet been replaced by the Animal Health Law and remains in force for the time being, no adjustments to Austrian legislation are likely to be necessary, as it is in any case aligned with the Commission Regulation (EC) No. 1266/2007.

In conclusion, the authors assume that the Animal Health Law will be beneficial for Austria and many other countries with satisfactory epidemiological situations and/or already implemented mitigation activities against these four cattle diseases.

## Data Availability Statement

The original contributions presented in the study are included in the article/Supplementary Material, further inquiries can be directed to the corresponding author/s.

## Author Contributions

FFR and BC conceived, designed, coordinated the study, performed the data collection, drafted the manuscript, and approved the final manuscript. Data analysis and preparation of figures was done by FFR. Both authors contributed to the article and approved the submitted version.

## Conflict of Interest

The authors declare that the research was conducted in the absence of any commercial or financial relationships that could be construed as a potential conflict of interest.

## Footnotes

^1^Categories: ^1^ C+D+E, ^2^ D+E, ^3^ E.
