# Corrigendum: Overview of Mitigation Programs for Cattle Diseases in Austria

**DOI:** 10.3389/fvets.2021.822386

**Published:** 2021-12-23

**Authors:** Franz-Ferdinand Roch, Beate Conrady

**Affiliations:** ^1^Department for Farm Animals and Veterinary Public Health, Institute of Food Safety, Food Technology and Veterinary Public Health, University of Veterinary Medicine, Vienna, Austria; ^2^Department of Veterinary and Animal Sciences, Faculty of Health and Medical Sciences, University of Copenhagen, Frederiksberg, Denmark; ^3^Complexity Science Hub Vienna, Vienna, Austria

**Keywords:** animal health law, bluetongue, bovine viral diarrhea, enzootic bovine leucosis, eradication, control program, infectious bovine rhinotracheitis/infectious pustular vulvovaginitis

In the original article, there was an error. We used the phrase “non-regulated” for cattle diseases that are in fact listed in the New Animal Health Law that went into force in 2021.

A correction has been made to the title:

“Overview of mitigation programs for cattle diseases in Austria”

A correction has been made to the short running title:

“Austrian cattle disease control programs”

Corrections have been made to the **Abstract**, page 1, paragraph one:

“The non-mandatory regulation of animal diseases at the European Union (EU) level enables member states to implement mitigation programs based on their own country-specific conditions such as priority settings of the governments, availability of financial resources, and epidemiological situation.”

“This article aims to describe the past, current, and future mitigation activities and associated prevalence levels for four animal diseases, i.e., enzootic bovine leukosis (EBL), infectious bovine rhinotracheitis/infectious pustular vulvovaginitis (IBR/IPV), bovine viral diarrhea (BVD), and bluetongue disease (BT) for Austria.”

“The study results presented here are intended to contribute to a better comparison of the eradication status across European countries for cattle diseases by providing information about the mitigation activities and data of testing results over a period of 40 years.”

Corrections have been made to the **Introduction**, page 2, paragraph two, paragraph three:

“Besides the diseases listed in categories A and B, for which Union-wide regulations are implemented, there are animal diseases with no or limited mandatory regulations listed in categories C–E such as bluetongue disease^1^ (BT), epizootic hemorrhagic disease^2^, anthrax^2^, surra^2^, paratuberculosis^3^, Q fever^3^, infectious bovine rhinotracheitis/infectious pustular vulvovaginitis^1^ (IBR/IPV), bovine viral diarrhea^1^ (BVD), bovine genital campylobacteriosis^2^, trichomonosis^2^, and enzootic bovine leukosis^1^ (EBL) (2).”

“No or limited mandatory regulation of these cattle diseases at the EU level enables researchers to implement mitigation programs based on country-specific conditions such as priority settings of the governments, availability of financial resources, epidemiological situation such as the level of prevalence, and the importance of export for the national economy.”

A correction has been made to the **Materials and Methods** section, page 2, paragraph two:

“All data collected for the four cattle diseases, i.e., EBL, IBR/IPV, BVD, and BT, for Austria regarding the number of tested animals, tested bulk milk, positively tested animals, affected livestock, and changes in the sample size associated with changes of law over a 40-year period are provided in **Figures 2–8** and in **Supplementary Tables 1–3** and **Supplementary Figures 1, 2**.”

A correction has been made to the **Results** section, sub-section “*Bovine Viral Diarrhea”*, page 6, paragraph one:

“BVDV is an important infectious agent in the cattle population and has a global economic impact both through production losses such as reproductive dysfunction and costs of mitigation activities (53–59).”

The authors apologize for these errors and state that they do not change the scientific conclusions of the article in any way. The original article has been updated.

## Publisher's Note

All claims expressed in this article are solely those of the authors and do not necessarily represent those of their affiliated organizations, or those of the publisher, the editors and the reviewers. Any product that may be evaluated in this article, or claim that may be made by its manufacturer, is not guaranteed or endorsed by the publisher.

## Footnotes

^1^Categories: ^1^ C+D+E, ^2^ D+E, ^3^ E.

