# Supplementary material for: Overview of Mitigation Programs for Cattle Diseases in Austria
Source: Front Vet Sci. 2021 Jun 15;8:689244. doi: 10.3389/fvets.2021.689244 (PMC8239179; doi:10.3389/fvets.2021.689244)
Supplement: Supplementary file 1 [file Data_Sheet_1.docx]

**Resources of federal BVD data:**

Styria:

<https://www.verwaltung.steiermark.at/cms/ziel/79958253/DE/>

Upper Austria:

<https://www.ooe-tgd.at/454.htm>

Lower Austria:

<http://www.lkv-service.at/MEDIA/LKV_Kennzahl_September_BVD.pdf>

Tyrol and Vorarlberg:

<https://verbraucherschutz.sachsen-anhalt.de/fileadmin/Bibliothek/Politik_und_Verwaltung/MS/LAV_Verbraucherschutz/veterinaermedizin/veranstaltungen/symposium_fb4/neuntes/16_Schoepf.pdf>

**Referenced Austrian legislation:**

N.B. the versions of the referenced laws and ordinances depend on the cited statement and are not necessarily the latest versions. The latest version of each law and ordinance (or their successors) in this list are further referenced below and can be connected with this list by [RL + a consecutive number]. “=” means that the specified version corresponds to the referenced latest version. All referenced legislation is available from: https://www.ris.bka.gv.at/.

AL1. AustrianLaw. Bundesgesetz vom 1. Juni 1982 zur Bekämpfung der enzootischen Rinderleukose (Rinderleukosegesetz) BGBl. Nr. 272/1982. 1982. In the version of 1 June 1982 [RL1]

AL2. AustrianLaw. Bundesgesetz vom 26. Juni 1957 zur Bekämpfung der Brucellose (Abortus Bang) der Haustiere (Bangseuchen-Gesetz). StF: BGBl. Nr. 147/1957. 1957. In the version of 26 June [RL1]

AL3. AustrianLaw. Verordnung des Bundesministers für Gesundheit über die Programme zur Überwachung bestimmter Krankheiten bei Rindern (Rindergesundheits-Überwachungs-Verordnung) BGBl. II Nr. 334/2013. 2013. In the version of 10 March 2021 [=RL1]

AL4. AustrianLaw. Verordnung der Bundesministerin für Frauenangelegenheiten und Verbraucherschutz über die periodischen Untersuchungen von Rindern auf enzootische Rinderleukose (Rinderleukose-Untersuchungsverordnung) BGBl. II 443/1999. 1999. In the version of 30 November 1999 [RL1]

AL5. AustrianLaw. Bundesgesetz, mit dem das Tierseuchengesetz, das Tierarzneimittelkontrollgesetz, das Bangseuchen-Gesetz, das Rinderleukosegesetz, das IBR/IPV-Gesetz und das Bienenseuchengesetz geändert werden (Veterinärrechtsänderungsgesetz 2005) BGBl. I Nr. 67/2005. 2005. In the version of 5 July 2005 [RL1]

AL6. AustrianLaw. Verordnung des Bundesministers für Gesundheit über die Programme zur Überwachung bestimmter Krankheiten bei Rindern (Rindergesundheits-Überwachungs-Verordnung) BGBl. II Nr. 334/2013. 2013. In the version of 6 November 2013 [RL1]

AL7. Official Veterinary Bulletin of Austria. Kundmachung des Stichprobenplans für die blut- und milchserologische Untersuchung von Rindern auf Bangseuche, enzootische Rinderleukose und IBR/IPV gemäß § 18 Abs. 1 der Rindergesundheits-Überwachungs-Verordnung, BGBl. II Nr. 334/2013 GZ. 74.6000/0228-II/B/10/2013. 2013

AL8. AustrianLaw. Bekämpfung der Infektiösen Bovinen Rhinotracheitis und der Infektiösen Pustulösen Vulvovaginitis (IBR/IPV-Gesetz) BGBl. Nr. 636/1989. 1989. In the version of 28 December 1989 [RL1]

AL9. AustrianLaw. Regelung der zeitlichen Abstände zwischen den einzelnen periodischen Untersuchungen auf IBR/IPV BGBl Nr. 493/1991. 1991. In the version of 11 September 1991 [RL1]

AL10. AustrianLaw. Regelung der zeitlichen Abstände zwischen den einzelnen periodischen Untersuchungen auf IBR/IPV BGBl. Nr. 573/1996. 1996. In the version of 22 October 1996 [RL1]

AL11. AustrianLaw. Verordnung der Bundesministerin für Frauenangelegenheiten und Verbraucherschutz über die periodischen Untersuchungen von Rindern auf IBR/IPV (IBR/IPVUntersuchungsverordnung) BGBl. II Nr. 296/1999. 1999. In the version of 31 August 1999 [RL1]

AL12. AustrianLaw. Verordnung der Bundesministerin für Gesundheit, Familie und Jugend über die Untersuchung von Milch- und Blutproben zur Feststellung der IBR/IPV bei Rindern (IBR/IPV-Untersuchungsverordnung 2007) BGBl. II Nr. 306/2007. 2007. In the version of 5 November 2007 [RL1]

AL13. AustrianLaw. Verordnung der Bundesministerin für Gesundheit und Frauen über ein Untersuchungsprogramm zur Bekämpfung der Bovinen Virusdiarrhöe und der Mucosal Disease bei Rindern (BVD-Verordnung) BGBl. II Nr. 303/2004. 2004. In the version of 22 July 2004 [RL2]

AL14. AustrianLaw. Verordnung der Bundesministerin für Gesundheit, Familie und Jugend über ein Untersuchungsprogramm zur Bekämpfung der Bovinen Virusdiarrhöe und der Mucosal Disease bei Rindern (BVD-Verordnung 2007) BGBl. II Nr. 178/2007. 2007. In the version of 10 March 2021 [=RL2]

AL15. AustrianLaw. Verordnung der Bundesministerin für Arbeit, Soziales, Gesundheit und Konsumentenschutz über die Änderung der BVD-Verordnung 2007 (BVD-Verordnung-Novelle 2018) BGBl. II Nr. 92/2018. 2018. In the version of 7 May 2018 [RL2]

AL16. AustrianLaw. Verordnung des Bundesministers für Gesundheit, Sport und Konsumentenschutz über die Pflicht zur Anzeige von bestimmten, im Tierseuchengesetz nicht genannten Tierseuchen (Tierseuchen-Anzeigepflichtverordnung) BGBl. Nr. 756/1993. 1993. In the version of 9 November 1993 [RL3]

AL17. AustrianLaw. Gesetz vom 6. August 1909, betreffend die Abwehr und Tilgung von Tierseuchen (Tierseuchengesetz – TSG). StF: RGBl. Nr. 177/1909. 1909. In the version of 10 March 2021 [=RL3]

AL18. AustrianLaw. Verordnung des Bundesministers für Gesundheit und Konsumentenschutz über die veterinärbehördliche Grenzkontrolle und über das innergemeinschaftliche Verbringen von Tieren, Waren und Gegenständen (Veterinärbehördliche Einfuhr- und Binnenmarkt. Verordnung 1996; EBVO 1996) BGBl. Nr. 647/1996. 1996. In the version of 29 November 1996 [RL3]

AL19. AustrianLaw. Verordnung der Bundesministerin für Gesundheit, Familie und Jugend über ein Programm zur Überwachung der Freiheit von der Blauzungenkrankheit (Bluetongue-Überwachungsverordnung, BTÜ-V) BGBl. II Nr. 158/2007. 2007. In the version of 4 July 2007 [RL4]

AL20. AustrianLaw. Verordnung der Bundesministerin für Gesundheit und Frauen über Schutz- und Tilgungsmaßnamen zur Bekämpfung der Blauzungenkrankheit (Bluetongue-Bekämpfungsverordnung, BTB-V) BGBl. II NR. 515/2006. 2006. In the version of 27 December 2006 [RL5]

AL21. AustrianLaw. Verordnung der Bundesministerin für Gesundheit, Familie und Jugend zur Änderung der Bluetongue-Bekämpfungsverordnung (BTB-V-Änderungsverordnung) BGBl. II Nr. 250/2007. 2007. In the version of 20 September 2007 [RL5]

AL22. AustrianLaw Verordnung der Bundesministerin für Gesundheit, Familie und Jugend zur zweiten Änderung der Bluetongue-Bekämpfungsverordnung (2.BTB-V-Änderungsverordnung) BGBl. II Nr. 280/2007. 2007. In the version of 12 October 2007 [RL5]

AL23. AustrianLaw. Verordnung der Bundesministerin für Gesundheit, Familie und Jugend zur dritten Änderung der Bluetongue-Bekämpfungsverordnung (3. BTB-V-Änderungsverordnung) BGBl. II Nr. 293/2007. 2007. In the version of 24 October 2007 [RL5]

AL24. AustrianLaw. Verordnung der Bundesministerin für Gesundheit, Familie und Jugend zur vierten Änderung der Bluetongue-Bekämpfungsverordnung (4. BTB-V-Änderungsverordnung) BGBl. II Nr. 346/2007. 2007. In the version of 7 December 2007 [RL5]

AL25. AustrianLaw. Verordnung der Bundesministerin für Gesundheit, Familie und Jugend zur Änderung der Bluetongue-Bekämpfungsverordnung und Festlegung von Impfgebieten (Änderung der Bluetongue-Bekämpfungsverordnung und Festlegung von Impfgebieten) BGBl. II Nr. 267/2008. 2008. In the version of 24 July 2008 [RL5]

AL26. AustrianLaw. Verordnung der Bundesministerin für Gesundheit, Familie und Jugend zur Änderung der Anhänge der Bluetongue-Bekämpfungsverordnung (Änderung der Anhänge der Bluetongue-Bekämpfungsverordnung) BGBl. II Nr. 383/2008. 2008. In the version of 30 October 2008 [RL5]

AL27. AustrianLaw. Verordnung der Bundesministerin für Gesundheit, Familie und Jugend zur Änderung der Anhänge der Bluetongue-Bekämpfungsverordnung (Änderung der Anhänge der Bluetongue-Bekämpfungsverordnung) BGBl. II Nr. 396/2008. 2008. In the version of 18 November 2008 [RL5]

AL28. AustrianLaw. Verordnung des Bundesministers für Gesundheit, mit der die Bluetongue-Bekämpfungsverordnung geändert wird (Änderung der Bluetongue-Bekämpfungsverordnung) BGBl. II Nr. 240/2009. 2009. In the version of 24 July 2009 [RL5]

AL29. AustrianLaw. Verordnung der Bundesministerin für Gesundheit, mit der die Bluetongue-Bekämpfungsverordnung 2013 geändert wird (Änderung der Bluetongue-Bekämpfungsverordnung 2013) BGBl. II Nr. 362/2015. 2015. In the version of 20 November 2015 [RL5]

AL30. AustrianLaw. Verordnung der Bundesministerin für Gesundheit, mit der die Bluetongue-Bekämpfungsverordnung 2013 geändert wird (Änderung der Bluetongue-Bekämpfungsverordnung 2013) BGBl. II Nr. 428/2015. 2015. In the version of 20 November 2015 [RL5]

AL31. AustrianLaw. Verordnung der Bundesministerin für Gesundheit und Frauen, mit der die Verordnung über Schutz- und Tilgungsmaßnahmen zur Bekämpfung der Blauzungenkrankheit (Bluetongue-Bekämpfungsverordnung 2013) geändert wird (Änderung der Bluetongue-Bekämpfungsverordnung 2013) BGBl. II Nr. 108/2017. 2017. In the version of 11 April 2017 [RL5]

AL32. AustrianLaw. Verordnung der Bundesministerin für Arbeit, Soziales, Gesundheit und Konsumentenschutz, mit der die Verordnung über Schutz- und Tilgungsmaßnahmen zur Bekämpfung der Blauzungenkrankheit (Bluetongue-Bekämpfungsverordnung 2013) geändert wird (Änderung der Bluetongue-Bekämpfungsverordnung 2013) BGBl. II Nr. 95/2018. 2018. In the version of 9 May 2018 [RL5]

AL33. AustrianLaw. Verordnung der Bundesministerin für Arbeit, Soziales, Gesundheit und Konsumentenschutz, mit der die Verordnung über Schutz- und Tilgungsmaßnahmen zur Bekämpfung der Blauzungenkrankheit (Bluetongue-Bekämpfungsverordnung 2013) geändert wird (Änderung der Bluetongue-Bekämpfungsverordnung 2013) BGBl. II Nr. 37/2019. 2019. In the version of 4 February 2019 [RL5]

AL34. AustrianLaw. Verordnung des Bundesministers für Gesundheit über Schutz- und Tilgungsmaßnahmen zur Bekämpfung der Blauzungenkrankheit (Bluetongue-Bekämpfungsverordnung 2013, BTB-VO 2013) BGBl. II Nr. 287/2013. 2013. In the version of 10 March 2021 [=RL5]

**Recent versions of the cited legislation above:**

RL1. AustrianLaw. Verordnung des Bundesministers für Gesundheit über die Programme zur Überwachung bestimmter Krankheiten bei Rindern (Rindergesundheits-Überwachungs-Verordnung) BGBl. II Nr. 334/2013. 2013. In the version of 10 March 2021

RL2. AustrianLaw. Verordnung der Bundesministerin für Gesundheit, Familie und Jugend über ein Untersuchungsprogramm zur Bekämpfung der Bovinen Virusdiarrhöe und der Mucosal Disease bei Rindern (BVD-Verordnung 2007) BGBl. II Nr. 178/2007. 2007. In the version of 10 March 2021

RL3. AustrianLaw. Gesetz vom 6. August 1909, betreffend die Abwehr und Tilgung von Tierseuchen (Tierseuchengesetz – TSG). StF: RGBl. Nr. 177/1909. 1909. In the version of 10 March 2021

RL4. AustrianLaw. Verordnung der Bundesministerin für Gesundheit, Familie und Jugend über ein Programm zur Überwachung der Freiheit von der Blauzungenkrankheit (Bluetongue-Überwachungsverordnung, BTÜ-V) BGBl. II Nr. 158/2007. 2007. In the version of 11 March 2021

RL5. AustrianLaw. Verordnung des Bundesministers für Gesundheit über Schutz- und Tilgungsmaßnahmen zur Bekämpfung der Blauzungenkrankheit (Bluetongue-Bekämpfungsverordnung 2013, BTB-VO 2013) BGBl. II Nr. 287/2013. 2013. In the version of 10 March 2021


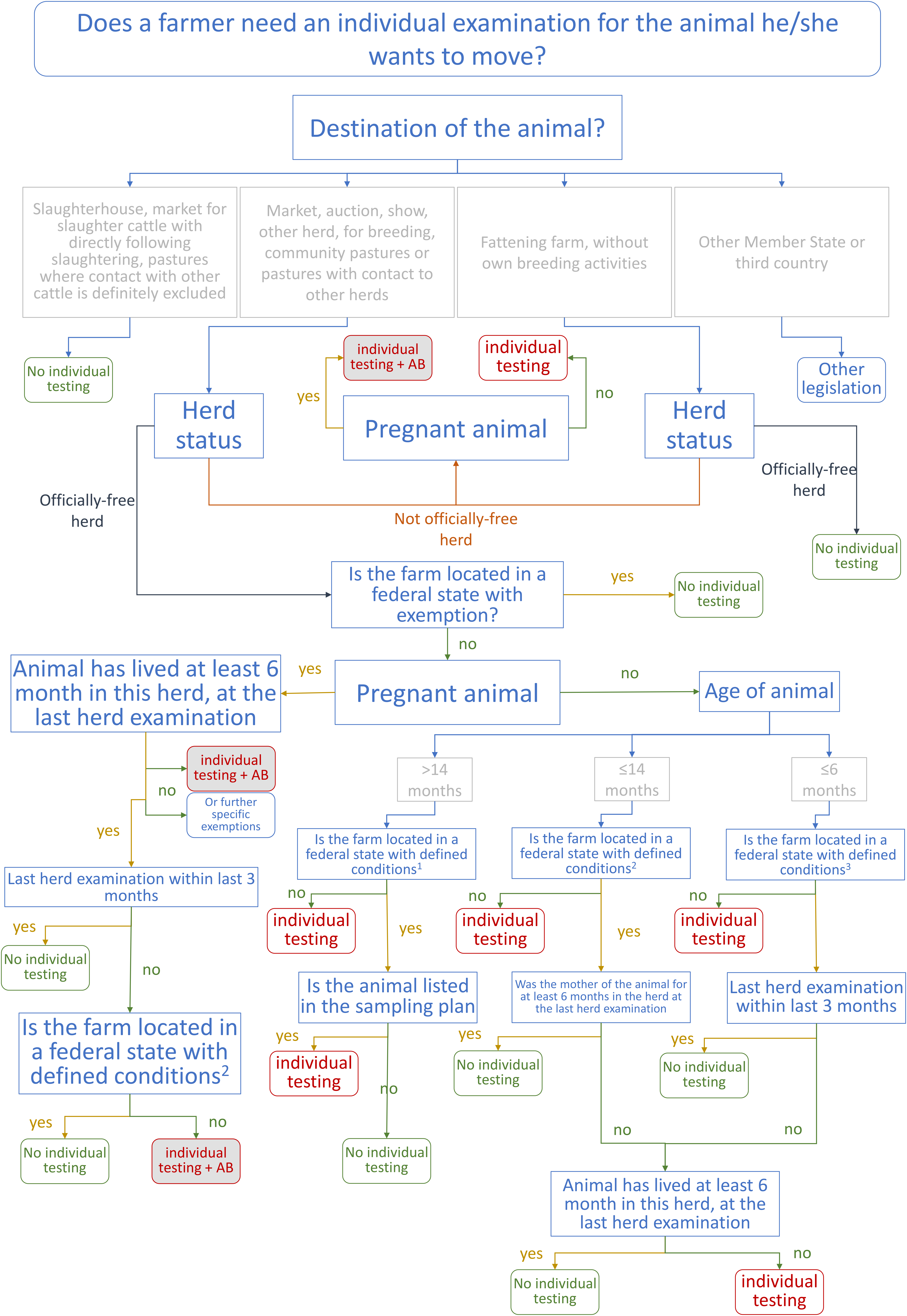


Figure S1: Simplified decision tree about whether an animal needs an individual testing regarding persistent infection (PI), if it should be moved^1^those are i) no new BVD cases in this federal state within the last two years, ii) at least 95% of the holdings in this federal state are officially BVD-free, iii) the control testing were done properly until the beginning of the testing, and iv) the federal state has a proper surveillance programme^2^those are i) the federal state proofed progress in the mitigation process, ii) new BVD outbreaks in less than 0.025% of the holdings in this federal state, iii) at least 95% of the holdings in this federal state are officially BVD-free^3^ those are i) the federal state proofed progress in the mitigation process, and ii) the number of new BVD outbreaks is constantly low. N.B. the superscript numbers in the caption here correspond to the superscript numbers in the figure S1.

Table S1: EBL relevant data extracted from the Green Report and Annual Veterinary Report

| year | evolution of law | cattle >2 years^5)^ | tested animals | tested bulk milk samples | Positively tested animals | affected livestock |
| --- | --- | --- | --- | --- | --- | --- |
| 1978^1)^ | - | - | - | - | - | - |
| 1979 | voluntary eradication | 1,130,100 | NA^6)^ | - | 185 | - |
| 1980 |  | 1,129,100 |  |  |  |  |
| 1981 | - | 1,130,400 | - | - | - | - |
| 1982 |  | 1,125,400 | - | - | - | - |
| 1983 | first nationwide legislation, all animals >2 years were tested periodically | 1,157,300 | 650,000 | - | 842 | 318 |
| 1984 |  | 1,158,938 | 600,000 | - | 693 | 184 |
| 1985 | major legislative change: farms specialised to fattening, were excluded, if they only buy animals from farms with disease-free status | 1,154,298 | 600,000 | - | 130 | 40 |
| 1986^2)^ |  | 1,147,796 | 590,000 | - | 65 | 28 |
| 1987 |  | 1,118,553 | 580,000 | - | 25 | 13 |
| 1988 |  | 1,101,101 | 560,000 | - | 26 | 14 |
| 1989 |  | 1,109,090 | 631,000 | - | 4 | 3 |
| 1990 |  | 1,097,949 | 574,667 | - | 4 | 3 |
| 1991 |  | 1,083,639 | 550,000 | - | 0 | 0 |
| 1992 |  | 1,048,035 | 550,000 | - | 0 | 0 |
| 1993 |  | 1,055,419 | 570,000 | - | 2 | 2 |
| 1994 |  | 1,048,762 | 481,914 | - | 0 | 0 |
| 1995 |  | 1,070,019 | 490,301 | - | 2 | 2 |
| 1996 |  | 1,064,144 | NA^6)^ | - | 1 | 1 |
| 1997 |  | 1,052,607 | 780,235 | - | 1 | 1 |
| 1998 |  | 1,040,409 | 377,857 | - | 0 | 0 |
| 1999^3)^ | major legislative change: all animals >2 years were tested in 20% of all holdings each year | 1,033,942 | 426,732 | - | 0 | 0 |
| 2000 |  | 1,033,595 | 232,112 | - | 1 | 1 |
| 2001 |  | 1,003,812 | 217,601 | - | 5 | 5 |
| 2002 |  | 976,950 | 213,635 | - | 0 | 0 |
| 2003 |  | 964,272 | 204,194 | - | 0 | 0 |
| 2004 |  | 962,648 | 196,010 | - | 0 | 0 |
| 2005 | major legislative change: bulk milk testing was possible | 945,951 | 205,374 | - | 0 | 0 |
| 2006 |  | 936,399 | 201,931 | - | 1 | 1 |
| 2007^4)^ |  | 929,049 | 146,515 | 14,797 | 0 | 0 |
| 2008 |  | 929,496 | 36,903 | 37,996 | 0 | 0 |
| 2009 |  | 936,857 | 27,366 | 35,973 | 0 | 0 |
| 2010 |  | 935,577 | 30,284 | 35,427 | 0 | 0 |
| 2011 |  | 923,233 | 30,295 | 33,596 | 0 | 0 |
| 2012 |  | 903,003 | 28,101 | 31,244 | 0 | 0 |
| 2013 | major legislative change: harmonization with IBR/IPV and Brucella abortus surveillance | 896,764 | 10,304 | 1,266 | 0 | 0 |
| 2014 |  | 899,343 | 12,281 | 1,403 | 0 | 0 |
| 2015 |  | 894,046 | 11,619 | 1,346 | 0 | 0 |
| 2016 |  | 890,198 | 11,778 | 1,288 | 0 | 0 |
| 2017 |  | 881,368 | 10,964 | 1,323 | 0 | 0 |
| 2018 |  | 866,207 | 10,244 | 1,281 | 0 | 0 |
| 2019 |  | 847,773 | - | - | 0 | 0 |
| 2020 |  | 840,807^7)^ | 10,300^8)^ | 1,211^9)^ | 0 | 0 |
| ^1)^ first evaluations | | | | | | |
| ^2)^ all states gained disease free status  ^3)^ official disease-free status according to | | | | | | |
| ^4)^ bulk milk testing was established nationwide | | | | | | |
| ^5)^  qualifying date 01 December of each year | | | | | | |
| ^6)^ number of tested animals not published | | | | | | |
| ^7)^ qualifying date 01 June 2020 | | | | | | |
| ^8)^ approximation | | | | | | |
| ^9)^ planned sample size for 2020 | | | | | | |
|  | | | | | | |

Table S2: IBR/IPV relevant data extracted from the Green Report and Annual Veterinary Report

| year | evolution of law | cattle >2 years | tested animals | tested bulk milk samples | Positively tested animals | affected livestock |
| --- | --- | --- | --- | --- | --- | --- |
| 1987^1)^ | - | - | - | - | - | - |
| 1988 | voluntary eradication | 1,101,101 | 1,500,000 | - | 9,000 | - |
| 1989 |  | 1,109,090 |  | - |  | - |
| 1990 | first nationwide legislation, all animals >2 years were tested periodically all 24-27 months | 1,097,949 | 593,000 | - | 1,989 | - |
| 1991 |  | 1,083,639 | 550,000 | - | 1,300 | - |
| 1992 |  | 1,048,035 | 550,000 | - | 468 | - |
| 1993 |  | 1,055,419 | 550,000 | - | 190 | 62 |
| 1994^2)^ |  | 1,048,762 | 478,876 | - | 72 | 63 |
| 1995^2)^ |  | 1,070,019 | 490,261 | - | 875 | 205 |
| 1996 | major legislative change: all animals >2 years were tested periodically all 12-15 months | 1,064,144 | NA^6)^ | - | 132 | 54 |
| 1997 |  | 1,052,607 | NA^6)^ | - | 847 | 91 |
| 1998 | major legislative change: bulk milk testing was possible | 1,040,409 | 736,252 | - | 344 | 68 |
| 1999^4)^ | major legislative change: sampling plan | 1,033,942 | 412,449 | - | 46 | 7 |
| 2000 |  | 1,033,595 | 109,403 | - | 214 | 9 |
| 2001 |  | 1,003,812 | 112,357 | - | 6 | 2 |
| 2002 |  | 976,950 | 107,427 | - | 4 | 4 |
| 2003 |  | 964,272 | 108,126 | - | 33 | 30 |
| 2004 |  | 962,648 | 96,782 | - | 17 | 16 |
| 2005 | minor legislative changes | 945,951 | 99,626 | - | 6 | 1 |
| 2006 |  | 936,399 | 108,038 | - | 3 | 2 |
| 2007^5)^ |  | 929,049 | 78,149 | 14,797 | 2 | 2 |
| 2008 |  | 929,496 | 29,391 | 37,996 | 4 |  |
| 2009 |  | 936,857 | 27,366 | 35,973 | - | 1 |
| 2010 |  | 935,577 | 30,333 | 35,428 | 0 | 0 |
| 2011 |  | 923,233 | 30,186 | 33,596 | 1 | 1 |
| 2012 |  | 903,003 | 28,204 | 31,244 | 0 | 0 |
| 2013 | major legislative change: harmonization with EBL and Brucella abortus surveillance | 896,764 | 10,296 | 1,266 | 0 | 0 |
| 2014 |  | 899,343 | 11,262 | 1,401 | 0 | 0 |
| 2015 |  | 894,046 | 48,382 | 7,400 | 313 | 26 |
| 2016 |  | 890,198 | 11,185 | 1,288 | 0 | 0 |
| 2017 |  | 881,368 | 11,659 | 1,547 | 0 | 0 |
| 2018 |  | 866,207 | 10,884 | 1,284 | 0 | 0 |
| 2019 |  | 847,773 | - | - | 0 | 0 |
| 2020 |  | 840,807^7)^ | 10,300^8)^ | 1,211^9)^ | 0 | 0 |
| ^1)^ first evaluations | | | | | | |
| ^2)^ all states gained disease free status | | | | | | |
| ^3)^ EU accession  ^4)^ additional guarantees | | | | | | |
| ^5)^ bulk milk testing was established nationwide | | | | | | |
| ^6)^ number of tested animals not published | | | | |  |  |

Table S3: BT relevant data extracted from the Annual Veterinary Report

| year | evolution of law | cattle^1)^ | no. serological tests^4)^ | no. PCR tests | positively tested animals | affected livestock |
| --- | --- | --- | --- | --- | --- | --- |
| 1998-2007 | only passive surveillance | 2,091,320^2)^ | - | - | 0 | 0 |
| 2007 | first nationwide legislation for surveillance (BTÜ-V) and control (BTB-V) | 2,000,196 | - | - | 0 | 0 |
| 2008 | BTB-V major legislative change: mandatory vaccination | 1,997,209 | 40,768^6)^ | 6,994^9)^ | 11 | 6 |
| 2009 | BTB-V major legislative change: voluntary vaccination, establishing of seasonally vector-free periods | 2,026,260 | 34,816^7)^ | 23,758^10)^ | 17 | 11 |
| 2010 |  | 2,013,281 | 32,054 | 37,469 | 0 | 0 |
| 2011 |  | 1,976,527 | 7,412^8)^ | 3,385 | 0 | 0 |
| 2012 |  | 1,955,618 | 1,245 | 38 | 0 | 0 |
| 2013 | BTB-V major legislative change: a stamp out of all susceptible animals is possible if necessary | 1,958,282 | 1,236 | 26 | 0 | 0 |
| 2014 |  | 1,961,201 | 1,228 | NA^5)^ | 0 | 0 |
| 2015 |  | 1,957,610 | 3,459 | 1,960 | 6 | 4 |
| 2016 |  | 1,954,391 | 5,589 | 180 | 4 | 3 |
| 2017 |  | 1,943,476 | 6,994 | 67 | 0 | 0 |
| 2018 |  | 1,912,808 | 7,025 | 47 | 0 | 0 |
| 2019 |  | 1,879,520 | - | - | - | - |
| 2020 |  | 1,844,335^3)^ | - | - | - | - |
| ^1)^qualifying date 01 December of each year | | | | | | |
| ^2)^mean | | | | | | |
| ^3)^qualifying date 01 June 2020 | | | | | | |
| ^4)^blood or milk | | | | | | |
| ^5)^number of samples not published | | | | | | |
| ^6)^additionally 3,038 other ruminants | | | | | | |
| ^7)^additionally 498 other ruminants | | | | | | |
| ^8)^other animals included, no separate numbers for cattle published | | | | | | |
| ^9)^additionally 1,346 other ruminants | | | | | | |
| ^10)^additionally 1434 other ruminants | | | | | | |


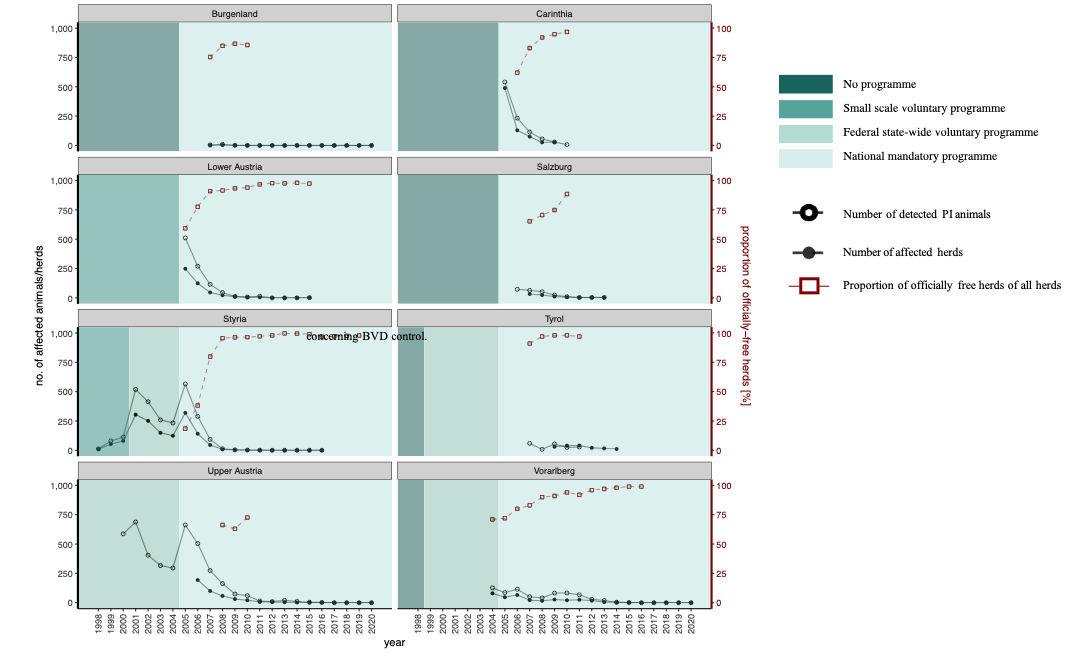


Figure S2: Data provided by Austrian federal states on personal request. Black dots represent the number of detected persistent infected (PI) animals (empty) and affected herds (filled). Red rectangles show the proportion of officially free herds of all cattle herds, subjected in the legislation concerning BVD control.
